# Himalayan black bulbuls (*Hypsipetes leucocephalus niggerimus*) exhibit sexual dichromatism under ultraviolet light that is invisible to the human eye

**DOI:** 10.1038/srep43707

**Published:** 2017-04-06

**Authors:** Hsin-Yi Hung, Carol K. L. Yeung, Kevin E. Omland, Cheng-Te Yao, Chiou-Ju Yao, Shou-Hsien Li

**Affiliations:** 1 Department of Life Science, National Taiwan Normal University, Taipei, 116, Taiwan; 2 Novogene Bioinformatics Institute, Beijing, 100083, P. R. China; 3 Department of Biological Sciences, University of Maryland, Baltimore County, 21250, USA; 4 Division of Zoology, Endemic Species Research Institute, Nantou County, 55244, Taiwan; 5 Department of Biology, National Museum of Natural Science, Taichung, 40453, Taiwan

## Abstract

Sexual dichromatism is a key proxy for the intensity of sexual selection. Studies of dichromatism in birds may, however, have underestimated the intensity and complexity of sexual selection because they used museum specimens alone without taking colour-fading into account or only measured conspicuous visual traits in live animals. We investigated whether the Himalayan black bulbul (*Hypsipetes leucocephalus nigerrimus*), which is sexually monomorphic to the human eye, exhibits sexual dichromatism distinguishable by a spectrometer. We measured the reflectance (within both the human visual perceptive and the ultraviolet ranges) of two carotenoid-based parts and eight dull and melanin-based parts for each individual live bird or museum skin sampled. According to an avian model of colour discrimination thresholds, we found that males exhibited perceptibly redder beaks, brighter tarsi and darker plumage than did females. This suggests the existence of multiple cryptic sexually dichromatic traits within this species. Moreover, we also observed detectable colour fading in the museum skin specimens compared with the live birds, indicating that sexual dichromatism could be underestimated if analysed using skin specimens alone.

One of the most robust and widely used indices of the intensity of sexual selection in birds is sexual dichromatism, in which males are typically brighter and more colourful or have more distinguishing features than females^1^,^2^. Although intersexual differentiation in mating behaviours, habitat preferences and predator avoidance could also have promoted the evolution of colouration, sexual dichromatism is considered to be driven mainly by female preferences or male-male competition^3^ for sexual recognition, individual quality assessment and sexual attraction^4^. However, several pitfalls in studies on sexual dichromatism may have led to an overall underestimation of the intensity of sexual selection in birds. Notably, these studies have mainly focused on conspicuous colour differences^5^,^6^,^7^ in the 400–700 nm range, which is perceptible to the human eye^8^,^9^. However, birds have a wider visual colour perception range (300–700 nm), and can detect ultraviolet (UV: 300–400 nm) colour differences^10^. Producing and maintaining UV colouration can be resource-intensive for birds^11^,^12^,^13^; therefore such colours can be used as a signal^14^,^15^ or a target^16^ for mate choice. With the aid of spectrometers, several avian species that were presumed to be monochromatic have been revealed to have dichromatic UV colouration^17^,^18^. Additional studies are, however, required for further evaluation of the extent of prevalence of UV dichromatism in birds.

Furthermore, examination of melanin-based colouration, which appears dull to humans but which may still carry signals of individual quality to birds given their superior vision, is underrepresented in avian sexual dichromatism studies^19^. Melanin-based characteristics are associated with an individual’s qualities, namely social rank, aggressive behaviour and immunocompetence, which are equally important as targets for sexual selection as are carotenoid-based characteristics^19^,^20^,^21^. Although the expression of both melanin- and carotenoid-based traits can be affected by an individual’s status^22^,^23^,^24^, melanin deposition is more significantly controlled by genes than carotenoid deposition^19^,^25^. The sexual selection pressures on these traits might differ from those on other types of traits existing in the same organism. In recent years, increasing attention has been paid to the relative contribution of the two pigment-based colouration systems within the same species^26^.

The use of museum skin specimens for avian colouration studies could also lead to the underestimation of sexual dichromatism because specimens’ feather colours fade over time. This colour degradation is species-dependent and also depends on when the specimen was collected^27^,^28^,^29^. It has been shown that colour fading is significant for museum skin specimens collected more than 50 years previously^29^, but the level of degradation in the colour of newly collected museum specimens has been controversial^27^,^28^,^29^.

In this study, we used a spectrometer to study sexual dichromatism in a passerine, the Himalayan black bulbul (*H. leucocephalus nigerrimus*). This species is sexually monomorphic to the human eye: both sexes are entirely covered with a melanin-based black plumage with grey patches on their wings and have a carotenoid-based red beak and tarsus^25^. We investigate whether intersexual differences in characteristics are perceptible to the bulbul itself according to the Vorobyev-Osorio colour discrimination model^30^,^31^, which is based on the avian tetrahedral colour space^32^. Both live birds and skin specimens were measured to compare colour differences between them. Research skins from two museums were used, so different specimen preservations methods may have been used.

In this paper, we show that sexual dichromatism exists in the Himalayan black bulbul and provide insights into the potential functional roles of melanin- and carotenoid-based characteristics in this species. Meanwhile, considerable colour fading was observed in the museum skin specimens collected less than five years previously, which raises concerns regarding the use of newly collected skin specimens for studies on avian cryptic sexual dichromatism.

## Results

**Figure 1:**
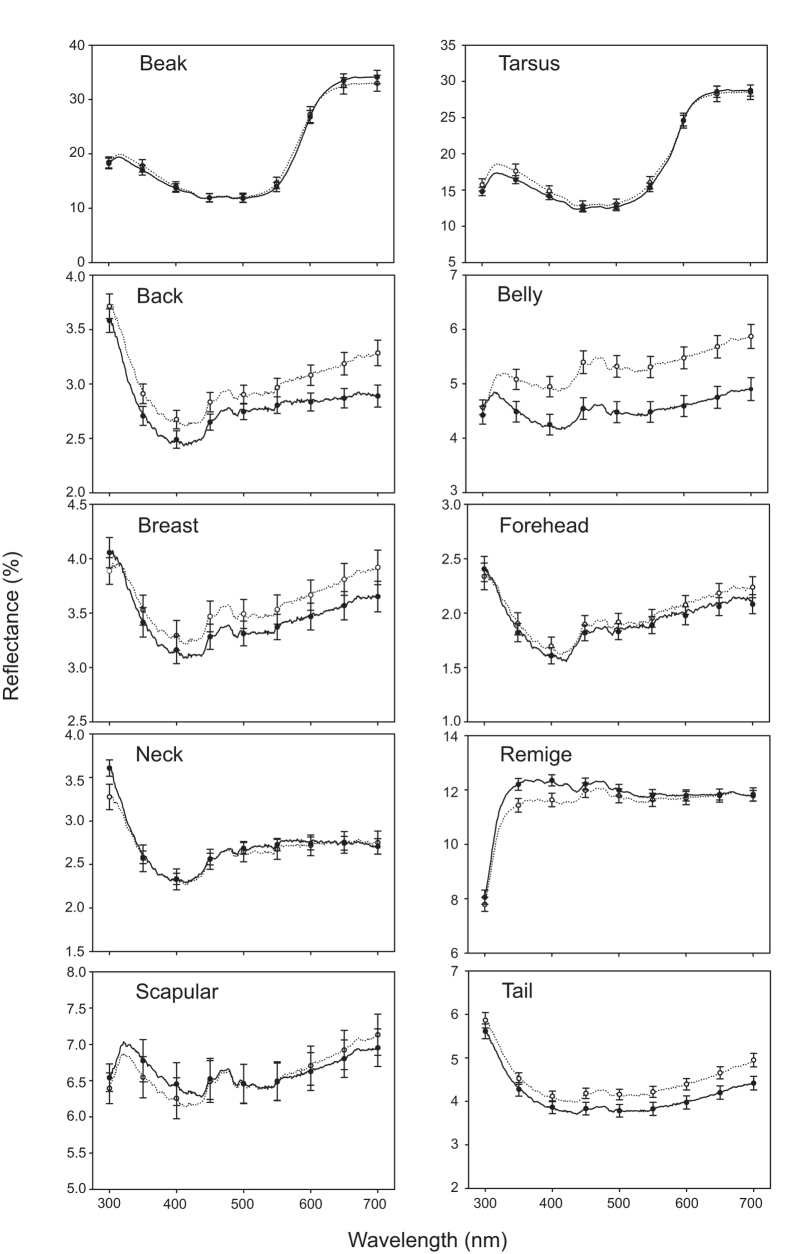
Spectra of ten characteristics in two sexes in live birds. Dotted lines indicate spectra of females (n = 57) and solid lines indicate females (n = 55).

The average spectra of the two sexes were nearly identical in appearance but varied in total reflectance (Fig. 1). The carotenoid-based evaluations for beaks and tarsi showed two peaks at wavelengths of 300–400 nm and 600–700 nm, which are the reflectance ranges of UV light and carotenoid-based feathers respectively. By contrast, the spectra for the melanin-based parts were almost flat but with a moderate rise in the UV section. In the live birds, the carotenoid-based parts differed significantly between the sexes (Table 1; whole model, *F* = 2.82, *p* = 0.01), specifically in beak hue, where males exhibited redder beaks than did females (Ls mean: males, 590.2 ± 0.6 nm and females, 587.9 ± 0.7, ANOVA with the covariate of year and the interaction factors of sex and year, *F* = 5.95, *p* = 0.016, Supplementary Fig. S1). No significant differences were observed between the sexes when considering the melanin-based parts (Table 1, whole model, *F* = 1.47, *p* = 0.136). A significant effect of study year was observed in both carotenoid- and melanin-based parts (Table 1). Melanin-based plumage in the museum specimens did not differ between the sexes (Table 1).

An analysis using the Vorobyev-Osorio colour discrimination model revealed more parts, namely carotenoid-based beak and tarsus and the melanin-based remige and tail, that were considerably dichromatic in live male and female birds (Table 2). In the museum skin specimens, in addition to the breast, the belly was also found to be sexually dichromatic. In summary, different subsets of the selected body parts were discovered to be sexually dichromatic in our live birds and in the museum skin specimens (Table 2).

Colour comparisons between the live birds and museum skin specimens showed significant colour fading in the latter (MANOVA, df = 15; *F*_*Sample type*_ = 6.35, *p* < 0.0001; *F*_*Sex*_ = 2.13, *p* = 0.01; *F*_*Sample type*×*Sex*_ = 1.57, *p* = 0.097). Live birds had brighter breasts and scapular feathers but lower brightness in the tail (Table 3a, Supplementary Table S1); they also had higher chroma_UV_ in every part (Table 3b, Supplementary Table S1). Different preservation methods or seasons of collection did not affect the colouration of skin specimens (MANOVA, museum: df = 15, *F* = 18.21, *p* = 0.182; season of collection: df = 15, *F* = 8.33, *p* = 0.266, Table 1). Although all skin specimens were collected less than 20 years previously, a weak effect of the age of specimens was observed (MANOVA, df = 30, *F* = 7.31, *p* = 0.033, Table 1), specifically in the scapular feather where older specimens were significantly less bright than more recent ones (specimens 15–10 years old 4.52 ± 0.99%, specimens 10–5 years old 5.13 ± 0.53%, specimens less than 5 years old 7.21 ± 0.69%; *F* = 3.70, *p* = 0.042, Supplementary Table S2).

Variability in colour brightness within females was nearly identical to that among males for all body parts (Table 4). Surprisely, the variability in the brightness of different individuals’ sexually dichromatic parts (carotenoid-based beak, tarsus and melanin-based remige and tail) was similar with those of the sexually monochromatic parts (Table 2, variances of sexually dichromatic traits 25.82 ± 2.52%, variances of non-sexually dichromatic traits 28.06 ± 2.06%, Two-way ANOVA with cofactor sex, *F* = 0.457, *p* = 0.500).

## Discussion

We have shown significant sexual dichromatism in both the carotenoid- and melanin-based body parts of the Himalayan black bulbul by considering both their reflectance and spectral shape. Males’ redder bills, brighter tarsi and darker plumage were significantly different enough from females’ for birds to distinguish between them. This provides an insight into this species’ mating behaviour. We also found colour degradation in museum samples, which could lead to different conclusions on sexual dichromatism when skin specimens or live birds are studied.

Feather colourations of the Himalayan black bulbul and most pycnonotids (members of Pycnonotidae), appear dull to humans and they are considered monomorphic^33^. However, our results suggest that the extent of their sexual dichromatism could be underestimated. The differences are not large but are distinguishable (Table 2; ΔS of Black bird (*Turdus merula*): 5.56–9.21; ΔS of Black cap (*Sylvia atricapilla*): 1.48–16.9; ΔS of Greenfinch (*Carduelis chloris*): 2.26–8.10)^34^, indicating the potential for moderate sexual selection. Like most pycnonotids, the Himalayan black bulbul is socially monogamous and provides bi-parental care^33^, [personal observation]. Dunn *et al*.^35^ analysed more than 1000 species of birds and observed lower sexual dimorphism in monogamous species than that observed in birds with polygynous or lekking mating systems, where the variance in male mating success is thought to be lower. Nevertheless, other aspects of the Himalayan black bulbul and related species’ reproductive biology may contribute to sexual dichromatism; these include the genetic mating system and the parental investment of each sex, which should be investigated further.

Where males are subject to female mate choice, their sexually selected traits are usually more variable than females^3^,^36^. The similar variability that we found in female and male black bulbuls’ sexually dichromatic traits suggests that mate choice might be mutual in this species. Whereas studies of sexual selection have mostly focused on female choice and male–male competition, data increasingly shows that males can be choosy and benefit from mating females whose reproductive potential is high^37^,^38^,^39^,^40^. Kokko and Johnstone^41^ suggested that high species-specific and high sex-specific mate-encounter rates, high cost of breeding (parental investment), low cost of mate searching and highly variable quality of the opposite sex could promote the evolution of choosiness and that the primary determinant of sex roles in mate choice is parental investment. According to this hypothesis, the sex with a high breeding cost (mortality during signalling and caring) should evolve to be choosy. The reproductive biology of the Himalayan black bulbuls is unclear, but research on pycnonotids suggests comparable parental care loads between the sexes, and their breeding success is generally low (8.3–15%^33^,^42^) while the rate of predation is high. As such, high breeding costs and a comparable load of parental care between the sexes might promote mutual selection in pycnonotids. This is similar to trends also found in mammals^43^.

In many animal species, sexual dichromatism is strong and almost complete; almost any part of the male and female can be distinguished visually (e.g., peacock, *Pavo cristatus*; Orchard Orioles, *Icterus spurius*). However, there are many other species in which sexual dichromatism is much more subtle, including the Himalayan black bulbul. Dichromatism is termed “cryptic” when the sexes appear similar to the human eye, but display subtle, but statistically significant differences that on average separate males and females e.g.^6^,^17^,^18^. Although sexual dichromatism can be functional^14^,^44^,^45^,^46^,^47^, and is often the object of female choice, one single subtle sexually dichromatic trait might not provide sufficient information about the carrier to be useful. Species may therefore evolve the use of multiple characteristics to evaluate conspecifics. Studies have shown that females may choose mates based on multiple sexual ornaments^48^,^49^; multiple ornaments provide females with diverse information at different stages of mate choice^50^, or function as redundant signals to improve the accuracy of mate assessment^51^,^52^. Our data shows that both carotenoid- and melanin-based characteristics are sexually dichromatic in Himalayan black bulbuls, and both could convey information about an individuals’ physical conditions^53^, [unpublished]. We here propose that in our species, individuals may use multiple cues in sexual selection.

According to the condition-dependent handicap model, sexually selected traits show larger variability than non-sexually selected traits^3^,^36^. However, in our study, brightness did not vary more in the sexually dichromatic parts of the Himalayan black bulbul (beak, tarsus, remige and tail) than in the sexually monochromatic parts, which was consistent with Delhey and Peters’ finding when considering six avian species^34^. When combined with the findings that males’ colouration was no more variable in males than females’, this suggests that differences in colour between sexes in our species might not be shaped by the forces of sexual selection alone. As well as being the object of female choice, sexually dichromatic traits have been proved to function in quality signaling^45^,^47^ and agonistic interactions in several avian species^14^,^44^,^46^.

Study year had a significant effect on both carotenoid- and melanin-based characteristics in live birds (see in Table 1). Sex ratios varied among years (Female/Male ratios are 0.86, 2.18 and 0.61 in 2008, 2009 and 2011 respectively), but as we treated sex as a cofactor in our analysis, it cannot explain the remaining effect. Several factors, such as varying sex ratios, different population or different environment among years, could have caused the effects. Studies have shown that the colour of melanin-based plumage can vary among geographic populations^54^ or according to the nutrition changes in environment^55^. Either of these factors, might have caused the colour variation among years in our study.

Different sets of sexually dichromatic parts were detected in the live birds and museum skin specimens, and significant degradation of colour - whether pigment-based or structural - was found in skin specimens, some of which had been preserved for less than 5 years. These results suggest that the use of skin specimens in avian colouration study may be error-prone, contradicting the previous findings indicating that melanin- and carotenoid-based skins colors remain unchanged for at least 50 years after preservation^56^. Conversely, our results corroborated the conclusion drawn in a study comparing live birds and skin specimens of long-tailed manakins that significant differences in colorimetric variables were attributable to the age of the specimens^27^. Their results are consistent with another study reporting UV colour degradation in preserved skin specimens of approximately 300 bird species throughout Europe and the USA^29^. Colour degradation could possibly be caused by the preservation process, preservation agents, specimen preparation, contamination or simply age^27^. Given that museum skin specimens are widely used in studies of avian colouration^57^, we suggest that skin specimen colour should be pre-tested against live birds to minimize the possible effects of colour fading; measurements obtained from skin samples should be corrected for age and/or preservation condition, and the results should be interpreted with greater caution than before.

## Methods

### Study Species

The Himalayan black bulbul (*H. leucocephalus nigerrimus*) is a subspecies endemic to Taiwan. It is widely distributed, inhabiting broadleaf, evergreen and mixed deciduous forests, groves, clearings and edges. It breeds monogamously from April to July. A total of 112 live individuals were bought from a pet-shop (San Xing Bird Shop, Taipei, Taiwan; 25.034398,121.504444) during the non-breeding seasons (mainly in November and December) in 2008, 2009 and 2011. These birds were all captured from the southern mountainous areas in Taiwan based on information provided by the pet shop owner. A blood sample was taken from each bulbul for molecular sex typing before proceeding to colour quantification. We also examined 37 research specimens from the archives of both Taiwan’s National Museum of Natural Science (five females and 11 males) and Endemic Species Research Institute (seven females and 14 males); all specimens had been collected within the previous 15 years. In both museums, all study skins are kept in the drawers in the archive room at constant temperature and humidity (20–25 °C and 40–45% in Taiwan’s National Museum of Natural Science, 20–22 °C and 50–55% in Endemic Species Research Institute).

### Molecular Sex Typing

Genomic DNA was extracted from the blood samples with traditional proteinase K digestion followed by a LiCl extraction^58^. The detailed program set-up of the polymerase chain reactions (PCRs) for molecular sex typing^59^ was the same as in Hung and Li^53^. In total, 55 male and 57 female live bulbuls were identified.

### Colour Measurement

For each individual, the reflectance of ten body parts, including two carotenoid-based parts, the beak and tarsus, and eight melanin-based parts, the forehead, nape, back, breast, belly, tail, remige and scapular feathers, were measured using a USB2000 spectrometer (Ocean Optics, Dunedin, FL, USA) with a HL2000 deuterium-halogen light source (Ocean Optics). The procedure for measurement was as described in Hung and Li^53^. Because of the obvious fading of carotenoid-based colouration, we did not score the colouration of beaks and tarsi in the museum specimens. We measured the colouration after verifying the absence of obvious stains or abrasions on the surface to reduce errors caused by diminished light reflectance. The data on melanin-based parts used in this study were extracted and reanalysed from Hung and Li^53^.

### Colour Quantification

We used a combination of colorimetric variables, namely hue, total brightness and chroma^60^, to quantify colouration of each characteristic of each individual. Hue was calculated for beaks and tarsi by identifying the wavelength of the mean of maximum and minimum reflectance values within a range 550–700 nm. Total brightness was calculated for all selected parts by averaging the reflectance observed within a range of 300–700 nm. Two kinds of chroma were calculated: Chroma_RED_ is the proportion of the reflectance for beak and tarsus within the range of 550–700 nm in the total brightness. Chroma_UV_ is the proportion of the reflectance for all parts within the range of 300–400 nm in the total brightness.

### Colour Discrimination

To distinguish between colorimetric variables for both sexes, we used multivariate analysis of variance (MANOVA) to compare the male and female average measurements while including the year of examination as a cofactor. MANOVA was used for the museum specimens with cofactors of the date of sample collection (in years) and the museums in which the samples were preserved. We also used the same statistical method to compare the same colorimetric variables between skin specimens and the live birds.

We calculated the variability (coefficients of variation) in brightness among individuals of each sex to examine whether the divergence of colour differences among females was different from that among males for the various body parts. We used two-way ANOVA for evaluating whether sexually dichromatic parts exhibited higher variability than others.

In addition, considering the differences in the spectral sensitivity of the four avian cone types, we mapped their spectra onto Goldsmith’s tetrahedral colour space system^32^ that has recently been recommended for analysing avian colouration^6^,^61^,^62^. We converted the spectral measurements into points within a tetrahedron, where the vertices correspond to exclusive stimulation of the ultraviolet (UV)-, blue (B)-, green (G)- and red (R)- sensitive cones in the avian eye. The quantum catch of each receptor was measured using the following equation:


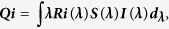


where λ denotes wavelength, *Ri(λ*) is the spectral sensitivity of the cone cell type *i (i* from 1–4 represent the four cone cells, UVS or VS, SWS, MWS and LWS respectively), *S(λ*) is the reflectance spectrum of a given feather patch, *I (λ*) is the irradiance spectrum entering the eye and integration is over the entire avian visual range (300–700 nm). The program Tetracolorspace^61^ was used for spectrum conversion, and the average spectral sensitivity curves of the UVS-type retinas^63^ were selected as the candidate avian vision in this study. After calculating the *Qi*, we calculated discriminability of colours in different body parts of each pair of average males and females in different body patches using the Vorobyev–Osorio colour discrimination model^30^,^31^. The model calculates the distance (ΔS) between two sexes, defined as the difference in the quantum catch of each receptor type (cone cell) in the avian retina^6^ between two sexes, in avian colour space. To calculate ΔS, we used the following formula:


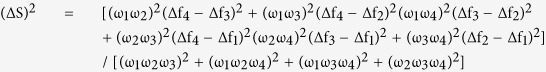


where ω_i_ is the constant noise-to-signal ratio (Weber fraction) for receptor type *i*, which in this study is based on empirical estimates obtained from the Pekin robin (*Leiothrix lutea*, ω_4_ = 0.05, following the ratio of the numbers of cones (UV: S: M: L = 1:2:2:4). Furthermore, f_i_ is proportional to the natural logarithm of the respective receptor quantum catches, which are normalized against an adapting background (according to the equations 2 and 3 in a study performed by Vorobyev *et. al*.^31^, and Δf_i_ is the difference between the signals in receptor i between two stimuli (two colours). When ΔS is below a threshold value of 1, colours were assumed to be indistinguishable.

### Ethics statement

Live birds were housed in the Animal Care House of the National Taiwan Normal University and cared for using procedures approved by the Institutional Animal Care and Use Committee of the Department of Life Science (IACUC Approval No 96026).

## Additional Information

**How to cite this article:** Hung, H.-Y. *et al*. Himalayan black bulbuls (*Hypsipetes leucocephalus niggerimus*) exhibit sexual dichromatism under ultraviolet light that is invisible to the human eye. *Sci. Rep.* **7**, 43707; doi: 10.1038/srep43707 (2017).

**Publisher's note:** Springer Nature remains neutral with regard to jurisdictional claims in published maps and institutional affiliations.

## Supplementary Material

Supplementary Information

## Figures and Tables

**Table 1:** Sexual dichromatism showed in carotenoid-based and melanin-based parts.

Parts tested	Item	df	*F*	*p*
Carotenoid-based parts				
	Year^a^	14	27.18	<0.0001*
	Sex	7	2.82	0.010*
	Sex*Year	14	1.29	0.216
Live melanin-based parts				
	Year	30	9.69	<0.0001*
	Sex	15	1.47	0.135
	Sex*Year	30	0.80	0.758
Skin specimen melanin-based parts				
	Museum^b^	15	18.21	0.182
	Sex	15	9.05	0.256
	Specimen age^c^	30	7.31	0.033*
	Season of colloection^d^	15	8.33	0.266

MANOVA.

^a^Three different sampling years, including 2008, 2009 and 2011.

^b^Skin specimens were collected from two museums, including Taiwan’s National Museum of Natural Science and Endemic Species Research Institute.

^c^Specimens were classified into three age categories, <5 yrs, 5–10 yrs and 10–15 yrs.

^d^The season collection of specimens were classified into two categories, breeding season (from April to July) and non-breeding season (from August to March).

**Table 2:** ΔS between the two sexes for live birds and skin specimens in different parts.

Parts	Live birds	Skin specimens
Beak	***3.15***	—
Tarsus	***2.28***	—
Back	0.28	0.88
Belly	0.64	***1.49***
Nape	0.08	0.61
Breast	0.09	***1.14***
Forehead	0.28	0.81
Remige	***1.19***	0.99
Scapular	0.66	***1.12***
Tail	***3.81***	0.52

ΔS > 1 is in bold and italics.

The beak and tarsus did not measure in skin specimens due to visible colour fading.

**Table 3:** Post-hoc test (Student’s t) of total brightness and chroma_UV_ between live birds and skin specimens after two-way ANOVA test (Supplementary Table S1).

(a) Total brightness				
Parts	Item	Mean* ± SE	Lower CL Difference	Upper CL Difference
Breast	Live	3.49 ± 0.08	0.24	1.00
	Skin	2.87 ± 0.17		
Scapular	Live	6.61 ± 0.19	0.41	2.11
	Skin	5.36 ± 0.38		
Tail	Live	4.23 ± 0.10	−1.44	−0.55
	Skin	5.22 ± 0.20		
**(b) Chroma**_**UV**_				
Back	Live	24.13 ± 0.19	1.57	3.29
	Skin	21.70 ± 0.39		
Belly	Live	23.13 ± 0.18	0.60	2.26
	Skin	21.70 ± 0.38		
Nape	Live	24.03 ± 0.23	2.19	4.20
	Skin	20.83 ± 0.45		
Breast	Live	24.10 ± 0.20	2.09	3.93
	Skin	21.09 ± 0.42		
Forehead	Live	23.39 ± 0.24	1.71	3.81
	Skin	20.63 ± 0.48		
Remige	Live	22.99 ± 0.17	0.07	1.64
	Skin	22.13 ± 0.36		
Scapula	Live	23.95 ± 0.16	0.95	2.36
	Skin	22.30 ± 0.32		
Tail	Live	25.79 ± 0.16	0.41	1.87
	Skin	24.65 ± 0.33		

*Least Square Mean, unit = %.

The Upper CL Difference and Lower CL Difference are the 95% confidence intervals for μ1 (Mean_live_) - μ2 (Mean_skin_).

**Table 4:** Variabilities (coefficients of variation, %) of brightness in the each sex among different parts.

Parts	Female	Male
Beak	37.43	38.76
Tarsus	29.53	24.50
Back	22.55	22.20
Belly	26.85	31.72
Nape	33.59	19.76
Breast	28.13	27.48
Forehead	32.55	31.79
Remige	14.84	12.12
Scapular feather	30.18	29.95
Tail	21.47	27.90

Chi-squared test. *P* < 0.05 is in bold and italics.

## References

[b1] Owens I. P. F. & Hartley I. R. Sexual dimorphism in birds: why are there so many different forms of dimorphism? P Roy Soc B 265, 397–407 (1998).

[b2] Seddon N. . Sexual selection accelerates signal evolution during speciation in birds. P Roy Soc B 280, 20131065 (2013). 10.1098/rspb.2013.1065 PMC3730587 23864596

[b3] Andersson M. B. Sexual selection (Princeton University Press, 1994).

[b4] Dale J. Intraspecific variation in coloration in *Bird coloration, function and evolution* (eds Hill G. E. & McGraw K. J.) Vol. 2, 36–86 (President and Fellows of Harvard College, 2006)

[b5] Bortolotti G. R., Negro J. J., Tella J. L., Marchant T. A. & Bird D. M. Sexual dichromatism in birds independent of diet, parasites and androgens. P Roy Soc B 263, 1171–1176 (1996).

[b6] Eaton M. D. Human vision fails to distinguish widespread sexual dichromatism among sexually monochromatic birds. P Natl Acad Sci USA 102, 10942–10946 (2005). 10.1073/pnas.0501891102 PMC1182419 16033870

[b7] Gray D. A. Carotenoids and sexual dichromatism in North American passerine birds. Am Nat 148, 453–480 (1996).

[b8] Cuthill I., Bennett A., Partridge J. & Maier E. Plumage reflectance and the objective assessment of avian sexual dichromatism. Am Nat 153, 183–200 (1999). 10.1086/303160 29578758

[b9] Neitz J. & Jacobs G. H. Polymorphism of the long-wavelength cone in normal human colour vision. Nature 323, 623- 625 (1986). 3773989 10.1038/323623a0

[b10] Chen D.-M., Collins J. S. & Goldsmith T. H. The ultraviolet receptor of bird retinas. Science 225, 337–340 (1984). 6740315 10.1126/science.6740315

[b11] Griggio M., Hoi H. & Pilastro A. Plumage maintenance affects ultraviolet colour and female preference in the budgerigar. Behav Process 84, 739–744 (2010). 10.1016/j.beproc.2010.05.003 20472042

[b12] Griggio M., Serra L., Licheri D., Campomori C. & Pilastro A. Moult speed affects structural feather ornaments in the blue tit. J Evol Biol 22, 782–792 (2009). 19320797 10.1111/j.1420-9101.2009.01700.x

[b13] Griggio M., Zanollo V. & Hoi H. UV plumage color is an honest signal of quality in male budgerigars. Ecol Res 25, 77–82 (2010).

[b14] Alonso-Alvarez C., Doutrelant C. & Sorci G. Ultraviolet reflectance affects male-male interactions in the blue tit (*Parus caeruleus ultramarinus*). Behav Ecol 15, 805–809 (2004).

[b15] Siefferman L. & Hill G. E. UV-blue structural coloration and competition for nestboxes in male eastern bluebirds. Anim Behav 69, 67–72 (2005).

[b16] Bennett A. T., Cuthill I. C., Partridge J. C. & Lunau K. Ultraviolet plumage colors predict mate preferences in starlings. P Natl Acad Sci USA 94, 8618–8621 (1997). 10.1073/pnas.94.16.8618 PMC23047 9238026

[b17] Igic B. . Size dimorphism and avian perceived sexual dichromatism in a New Zealand endemic bird, the whitehead *Mohoua albicilla*. J Morphol 271, 697–704 (2010). 20058295 10.1002/jmor.10827

[b18] Mays H. L. Jr . Sexual dichromatism in the yellow breasted chat *Icteria virens*: spectrophotometric analysis and biochemical basis. J Avian Biol 35, 125–134 (2004).

[b19] McGraw K. J. Mechanics of melanin-based coloration in *Bird Coloration: Function and evolution* (eds Hill G. E. & McGray K. J.) Vol. 2 243–295 (Harvard University Press, 2006)

[b20] Kingma S. A. . Sexual selection and the function of a melanin-based plumage ornament in polygamous penduline tits *Remiz pendulinus*. Behav Ecol Sociobiol 62, 1277–1288 (2008).

[b21] Tarof S. A., Dunn P. O. & Whittingham L. A. Dual functions of a melanin-based ornament in the common yellowthroat. P Roy Soc B 272, 1121–1127 (2005). 10.1098/rspb.2005.3053 PMC1559817 16024373

[b22] Thompson C. W., Hillgarth N., Leu M. & McClure H. E. High parasite load in house finches (*Carpodacus mexicanus*) is correlated with reduced expression of a sexually selected trait. Am Nat 149, 270–294 (1997).

[b23] Hill G. E. Proximate basis of variation in carotenoid pigmentation in male house finches. Auk 109, U1–12 (1992).

[b24] Nolan P. M., Hill G. E. & Stoehr A. M. Sex, size, and plumage redness predict house finch survival in an epidemic. P Roy Soc B 265, 961–965 (1998).

[b25] McGraw K. Mechanics of carotenoid-based coloration in *Bird Coloration: Function and evolution* (eds Hill G. E. & McGray K. J.) Vol. 2 177–242 (Harvard University Press, 2006).

[b26] Henschen A. E., Whittingham L. A. & Dunn P. O. Oxidative stress is related to both melanin-and carotenoid-based ornaments in the common yellowthroat. Func Ecol 30, 749–758 (2015).

[b27] Doucet S. M. & Hill G. E. Do museum specimens accurately represent wild birds? A case study of carotenoid, melanin, and structural colours in long-tailed manakins *Chiroxiphia linearis*. J Avian Biol 40, 146–156 (2009).

[b28] McNett G. D., Marchetti K. & Zink R. Ultraviolet degradation in carotenoid patches: live versus museum specimens of wood warblers (Parulidae). Auk 122, 793–802 (2005).

[b29] Pohland G. & Mullen P. Preservation agents influence UV-coloration of plumage in museum bird skins. J Ornithol 147, 464–467 (2006).

[b30] Vorobyev M. & Osorio D. Receptor noise as a determinant of colour thresholds. P Roy Soc B 265, 351–358 (1998). 10.1098/rspb.1998.0302 PMC1688899 9523436

[b31] Vorobyev M., Osorio D., Bennett A., Marshall N. & Cuthill I. Tetrachromacy, oil droplets and bird plumage colours. J Comp Phys A 183, 621–633 (1998). 10.1007/s003590050286 9839454

[b32] Goldsmith T. H. Optimization, constraint, and history in the evolution of eyes. Q Rev Biol 65, 281–322 (1990). 2146698 10.1086/416840

[b33] Fishpool L. D. C. & Tobias J. A. Family Pycnonotidae (Bulbuls) In Handbook of the Birds of the World, Vol 10 (eds Del Hoyo J., Elliot A. & Sargatal J.) Vol. 10, 124–250 (Lynx Editions, 2005).

[b34] Delhey K. & Peters A. Quantifying variability of avian colours: are signalling traits more variable? PLoS ONE 3, e1689 (2008). 18301766 10.1371/journal.pone.0001689 PMC2253496

[b35] Dunn P. O., Garvin J. C., Whittingham L. A., Freeman-Gallant C. R. & Hasselquist D. Carotenoid and melanin-based ornaments signal similar aspects of male quality in two populations of the common yellowthroat. Funct Ecol 24, 149–158 (2010).

[b36] Darwin C. The origin of species by means of natural selection: Or, the preservation of favoured races in the struggle for life and the descent of man and selection in relation to sex (Modern Library, 1872).

[b37] Edward D. A. & Chapman T. The evolution and significance of male mate choice. Trends Ecol Evol 26, 647–654 (2011). 21890230 10.1016/j.tree.2011.07.012

[b38] Jones K. M., Monaghan P. & Nager R. G. Male mate choice and female fecundity in zebra finches. Anim Behav 62, 1021–1026 (2001).

[b39] Amundsen T. & Forsgren E. Male mate choice selects for female coloration in a fish. P Natl Acad Sci USA 98, 13155–13160 (2001). 10.1073/pnas.211439298 PMC60840 11606720

[b40] Griggio M., Valera F., Casas A. & Pilastro A. Males prefer ornamented females: a field experiment of male choice in the rock sparrow. Anim Behav 69, 1243–1250 (2005).

[b41] Kokko H. & Johnstone R. A. Why is mutual mate choice not the norm? Operational sex ratios, sex roles and the evolution of sexually dimorphic and monomorphic signalling. Philost T Roy Soc B 357, 319–330 (2002). 10.1098/rstb.2001.0926 PMC1692955 11958700

[b42] Balakrishnan P. Reproductive biology of the square-tailed Black Bulbul *Hypsipetes ganeesa* in the Western Ghats, India. Indian Birds 5, 134–138 (2010).

[b43] Jones C. B. The Evolution of Mammalian Sociality by Sexual Selection In The Evolution of Mammalian Sociality in an Ecological Perspective (eds Vol. 8, 81–96 (Springer, 2014).

[b44] Bright A. & Waas J. R. Effects of bill pigmentation and UV reflectance during territory establishment in blackbirds. Anim Behav 64, 207–213 (2002).

[b45] Hill G. E. Redness as a measure of the production cost of ornamental coloration. Ethol Ecol Evol 8, 157–175 (1996).

[b46] Préault M., Deregnaucourt S., Sorci G. & Faivre B. Does beak coloration of male blackbirds play a role in intra and/or intersexual selection? Behav Process 58, 91–96 (2002). 10.1016/s0376-6357(02)00004-9 11955774

[b47] Walker L. K., Stevens M., Karadaş F., Kilner R. M. & Ewen J. G. A window on the past: male ornamental plumage reveals the quality of their early-life environment. P Roy Soc B 280, 2012–2852 (2013). 10.1098/rspb.2012.2852 PMC3574376 23407833

[b48] Chaine A. S. & Lyon B. E. Adaptive plasticity in female mate choice dampens sexual selection on male ornaments in the lark bunting. Science 319, 459–462 (2008). 18218896 10.1126/science.1149167

[b49] Doucet S. M. & Montgomerie R. Multiple sexual ornaments in satin bowerbirds: ultraviolet plumage and bowers signal different aspects of male quality. Behav Ecol 14, 503–509 (2003).

[b50] Borgia G. Complex male display and female choice in the spotted bowerbird: specialized functions for different bower decorations. Anim Behav 49, 1291–1301 (1995).

[b51] Johnstone R. A. Honest signalling, perceptual error and the evolution of’all-or-nothing’displays. P Roy Soc B 256, 169–175 (1994).

[b52] Moller A. & Pomiankowski A. Why have birds got multiple sexual ornaments? Behav Ecol Sociobio 32, 167–176 (1993).

[b53] Hung H.-Y. & Li S.-H. Brightness of melanin-based plumage coloration is a cue to oxidative stress in Himalayan Black Bulbuls (*Hypsipetes leucocephalus nigerrimus*). Avian Res 6, 1 (2015).

[b54] Argüelles-Ticó A. . Geographic variation in breeding system and environment predicts melanin-based plumage ornamentation of male and female Kentish plovers. Behav Ecol Sociobiol 70, 49–60 (2016). 26766883 10.1007/s00265-015-2024-8 PMC4701778

[b55] Wiebe K. L. & Vitousek M. N. Melanin plumage ornaments in both sexes of Northern Flicker are associated with body condition and predict reproductive output independent of age. Auk 132, 507–517 (2015).

[b56] Armenta J. K., Dunn P. O. & Whittingham L. A. Effects of specimen age on plumage color. Auk 125, 803–808 (2008).

[b57] Bridge E. S., Hylton J., Eaton M. D., Gamble L. & Schoech S. J. Cryptic plumage signaling in Aphelocoma scrub-jays. J Ornithol 149, 123–130 (2008).

[b58] Gemmell N. & Akiyama S. An efficient method for the extraction of DNA from vertebrate tissues. Trends Genet 12, 338–339 (1996). 8855658 10.1016/s0168-9525(96)80005-9

[b59] Fridolfsson A. K. & Ellegren H. A simple and universal method for molecular sexing of non-ratite birds. J Avian Biol 30, 116–121 (1999).

[b60] Montgomerie R. Analyzing colors in Bird coloration (eds Hill G. E. & McGraw K. J.) Vol. 1 90–147 (Harvard University Press, 2006).

[b61] Stoddard M. C. & Prum R. O. Evolution of avian plumage color in a tetrahedral color space: a phylogenetic analysis of new world buntings. Am Nat 171, 755–776 (2008). 18419340 10.1086/587526

[b62] Stoddard M. C. & Prum R. O. How colorful are birds? Evolution of the avian plumage color gamut. Behav Ecol 22, 1042–1052 (2011).

[b63] Endler J. & Mielke JR P. Comparing entire colour patterns as birds see them. Biol J Linn Soc 86, 405–431 (2005).

