# Supplementary material for: Himalayan black bulbuls (Hypsipetes leucocephalus niggerimus) exhibit sexual dichromatism under ultraviolet light that is invisible to the human eye
Source: Sci Rep. 2017 Apr 6;7:43707. doi: 10.1038/srep43707 (PMC5382547; doi:10.1038/srep43707)
Supplement: Supplementary Information [file srep43707-s1.pdf]

Himalayan black bulbuls (*Hypsipetes leucocephalus niggerimus*) exhibit sexual dichromatism under ultraviolet light that is invisible to the human eye

(SREP-16-43919A)

Hsin-Yi Hung, Carol K. L. Yeung, Kevin E. Omland, Cheng-Te Yao, Chiou-Ju Yao and Shou-Hsien Li*

Supplementary Figure S1 The colorimetric variables in carotenoid-based beak and tarsus between sexes in live birds. The hollow dots indicate males and the solid dots indicate females. Two-way ANOVA test, factor "Year" included three categories: 2008, 2009 and 2011, factor "Sex" included two categories: female and male.

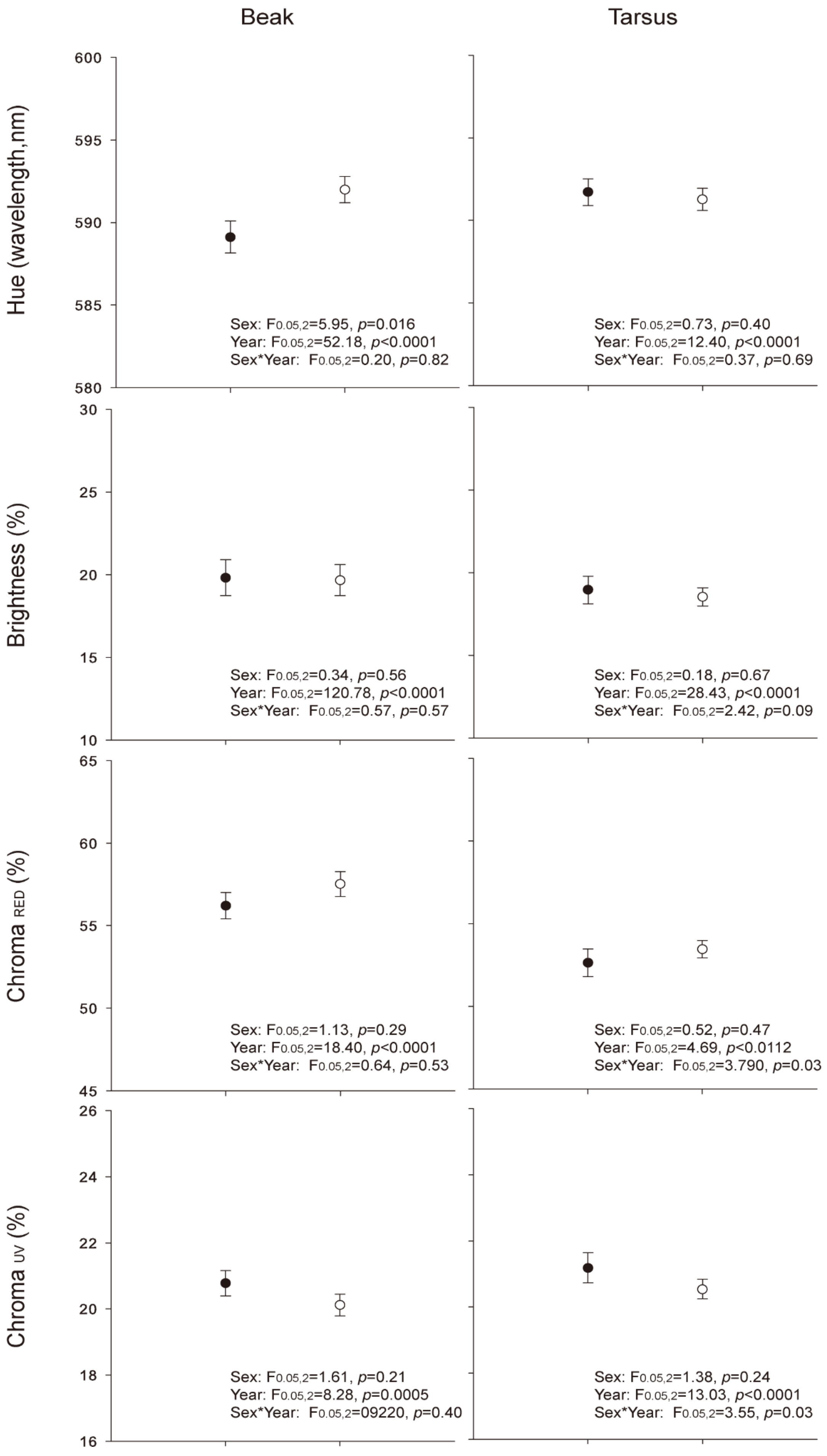

Supplementary Table S1 Two-way ANOVA of different melanin-based parts between live birds and skin specimens

Parts		Total brightness		$Chroma_{uv}$	
	Variables	*F*	*p*	*F*	*p*
Back	Item	2.11	0.15	31.24	**<.0001**
	Sex	2.48	0.12	8.95	**0.003**
	Item*Sex	2.78	0.10	8.93	**0.003**
Belly	Item	0.64	0.42	11.63	**0.001**
	Sex	7.18	**<0.001**	13.76	**<0.001**
	Item*Sex	0.02	0.90	1.05	0.31
Nape	Item	4.03	0.05	39.74	**<.0001**
	Sex	0.05	0.83	9.32	**0.003**
	Item*Sex	0.01	0.96	4.31	0.04
Breast	Item	10.30	**<0.002**	41.82	**<.0001**
	Sex	0.34	0.56	13.10	**0.0004**
	Item*Sex	0.10	0.75	7.63	0.007
Forehead	Item	0.01	0.94	26.81	**<.0001**
	Sex	0.17	0.68	9.41	**0.003**
	Item*Sex	0.72	0.40	5.53	0.02
Remige	Item	0.00	0.97	4.68	0.03
	Sex	2.77	0.10	2.16	0.14
	Item*Sex	0.19	0.66	0.09	0.76
Scapular	Item	8.64	**<0.004**	21.40	**<.0001**
	Sex	0.31	0.58	9.77	**0.002**
	Item*Sex	0.54	0.46	3.35	0.07
Tail	Item	19.52	**<.0001**	9.42	**0.003**
	Sex	1.97	0.16	2.30	0.13
	Item*Sex	0.10	0.76	0.06	0.81

Item: live bird and specimen, df= 1

Sex: female and male, df= 1

Item*sex: df=2.

Bold type indicates statistic significant (Bonferroin adjusted p = 0.005).

Supplementary Table S2 Two-way ANOVA of different melanin-based parts in skin specimens

Parts	Variables	Total brightness		$Chroma_{uv}$	
		F	*p*	*F*	*p*
Back	Sex	0.79	0.38	1.99	0.17
	Year	1.44	0.26	1.73	0.20
	Sex*Year	1.65	0.22	0.99	0.39
Belly	Sex	1.94	0.18	3.20	0.09
	Year	0.79	0.47	2.52	0.10
	Sex*Year	0.07	0.94	0.11	0.90
Nape	Sex	0.08	0.79	0.71	0.41
	Year	0.38	0.69	0.99	0.39
	Sex*Year	0.51	0.61	1.15	0.34
Breast	Sex	0.05	0.84	4.37	0.05
	Year	0.94	0.41	0.88	0.43
	Sex*Year	0.14	0.87	0.11	0.90
Forehead	Sex	0.00	0.97	1.47	0.24
	Year	0.09	0.91	1.26	0.30
	Sex*Year	2.37	0.12	0.73	0.50
Remige	Sex	0.22	0.65	0.15	0.70
	Year	3.14	0.06	0.11	0.90
	Sex*Year	0.69	0.52	0.05	0.95
Scapular	Sex	0.03	0.86	0.72	0.41
	Year	3.70	0.04	0.34	0.71
	Sex*Year	2.68	0.09	0.11	0.89
Tail	Sex	0.39	0.54	0.96	0.33
	Year	0.39	0.68	0.24	0.79
	Sex*Year	0.35	0.71	0.98	0.39

df: year-2, sex-1, sex*year-2

Year: indicating the years after making into skin specimens, including within 5yrs, 10yrs and 15yrs.

Bold type indicates statistic significant (Bonferroin adjusted p = 0.005).
